# Pharmacogenomic associations with HIV-1 virologic suppression in TB/HIV patients

**DOI:** 10.21203/rs.3.rs-5418156/v1

**Published:** 2024-12-16

**Authors:** Felipe Ridolfi, Gustavo Amorim, David W. Haas, Maria Arriaga, Cody Staats, Marcelo Cordeiro-Santos, Afrânio L. Kritski, Marina C. Figueiredo, Bruno B. Andrade, Timothy R. Sterling, Valeria C. Rolla, Regional Prospective Observational Research in Tuberculosis Brazil consortium (RePORT-Brazil)

**Affiliations:** Vanderbilt University Medical Center; Vanderbilt University Medical Center; Vanderbilt University Medical Center; Vanderbilt University Medical Center; Vanderbilt University Medical Center; Fundação Medicina Tropical Dr. Heitor Vieira Dourado; Universidade Federal do Rio de Janeiro; Vanderbilt University Medical Center; Instituto Gonçalo Moniz, Fundação Oswaldo Cruz; Vanderbilt University Medical Center; Instituto Nacional de Infectologia Evandro Chagas

**Keywords:** TB/HIV, genetic polymorphisms, HIV-1 treatment, virologic suppression, pharmacogenetic

## Abstract

**Background:**

Human genetic variants can affect TB and HIV drug metabolism, which may lead to toxicity or treatment failure. We evaluated associations between genetic variants of antiretroviral therapy (ART) and HIV-1 outcomes among TB/HIV patients.

**Methods:**

We included RePORT-Brazil participants with TB/HIV who initiated standard TB treatment [2 months of isoniazid/rifampicin (or rifabutin)/pyrazinamide/ethambutol, then 4 months or more of isoniazid/rifampicin (or rifabutin)], and ART. The endpoint was HIV-1 virologic suppression (defined as <1,000 HIV-1 RNA copies/mL, for primary analysis, and <50 HIV-1 RNA copies/mL, for secondary analysis) after at least 2 weeks of ART. We compared non-nucleoside reverse transcriptase inhibitor (NNRTI)-based and integrase strand transfer inhibitor (INSTI)-based ART regimens. We genotyped *CYP2B6* (rs3745274, rs28399499, rs4803419; affects efavirenz metabolism) and *UGT1A1* (rs887829; affects dolutegravir and raltegravir metabolism); all have defined normal, intermediate, and slow genotypes. Genotyping was performed by MassARRAY iPLEX Gold. We compared outcome proportions (Fisher’s test) and time-to-virologic suppression (survival analysis, Wilcoxon-Gehan test).

**Results:**

Among 194 TB/HIV participants included, efavirenz was the most frequent NNRTI ([n=76], one participant received etravirine), and raltegravir was the most frequent INSTI (n=88). The overall virologic suppression was suboptimal, with 32% (n=62) of participants not achieving HIV-1 virologic suppression. Among them, 36% (n=28) used efavirenz-based ART and were more likely to be *CYP2B6* normal metabolizers (n=8, 44%); and 30% (n=30) used INSTI-based ART and the *UGT1A1* normal genotype was also the most common (n=13, 50%). The median time to virologic suppression for efavirenz-based ART was 184 days (95% Confidence Interval (CI)160–207), and for INSTI-based ART, 188 days (95% CI 144–231) (p=0.84). No significant associations were found comparing the proportions and time to virologic suppression among *CYP2B6* and *UGT1A1*genotypes.

**Conclusions:**

In this observational cohort of patients treated for TB/HIV, the proportion of participants achieving virologic suppression was low, and genetic variants affecting ART metabolism were not significantly associated with the likelihood of virologic suppression.

## Introduction

Human Immunodeficiency Virus (HIV) infection is a risk factor for the development of tuberculosis (TB)^1–4^ and treatment of both disease is of high priority for TB/HIV co-infection management ^5,6^. However, TB and HIV regimens have drug-drug interactions and are also associated with toxicity^7^, which can impact the outcome of TB/HIV treatment in two ways: subtherapeutic concentrations can result in treatment failure and drug resistance, and supratherapeutic concentrations may be associated with treatment toxicity^8–10^. Moreover, the serum levels of some TB and HIV drugs can be influenced by single nucleotide polymorphisms (SNPs) of genes involved in the metabolism of these drugs^11–13^. Of the 25 antiretroviral therapy (ART) drugs approved by the Food and Drug Administration, nine (36%) are known to have SNPs associated with plasma exposure and/or side effects^14–25^. The proposed mechanisms of TB and HIV drug interactions are mainly related to substrate activity, particularly inhibition or induction of the hepatic system of cytochrome P450. Considering non-nucleoside reverse transcriptase inhibitors and integrase strand transfer inhibitors, the inducers of the enzymatic system (e.g., normal metabolizers) decrease serum drug concentrations, while inhibitors (e.g., slow metabolizers) increase the concentration^8^.

The dynamics between anti-TB drugs, ART, SNPs, and TB/HIV treatment outcomes are not yet fully understood. This study described the SNPs of the Brazilian population and evaluated the relationship between SNPs known to be associated with ART metabolism and HIV virologic suppression among TB/HIV participants in a large, prospective, cohort study in Brazil.

## Methods

### Study design and population

The Regional Prospective Observational Research in Tuberculosis (RePORT)-Brazil study enrolled participants with newly diagnosed, culture-confirmed, pulmonary TB at five sites across three regions in Brazil, between June 2015 and June 2019, and followed participants for two years. Sites were in Rio de Janeiro (Instituto Nacional de Infectologia Evandro Chagas, Clínica de Saúde Rinaldo Delmare, Secretaria de Saúde de Duque de Caxias), Salvador (Instituto Brasileiro para Investigação da Tuberculose), and Manaus (Fundação Medicina Tropical Dr. Heitor Vieira Dourado). The RePORT-Brazil population is broadly representative of TB cases in Brazil, as described previously.^26,27^ For this study, we included RePORT-Brazil participants with TB/HIV who initiated standard TB therapy and received ART during TB treatment.

### Variables and definitions

Clinical, demographic, and socio-economic data were collected longitudinally at baseline, month 2, and end of TB treatment visits, and during the follow-up period; for the latter, participants were contacted by telephone to assess signs and symptoms of TB recurrence.

The standard TB regimen was defined as a two-month intensive phase of isoniazid, rifampicin or rifabutin, pyrazinamide, and ethambutol, followed by a four-month (or more) continuation phase of isoniazid and rifampicin or rifabutin.^28^

All participants underwent HIV testing at baseline unless already known to be a person living with HIV/AIDS. We collected data on ART, CD4 cell count, and HIV-1 RNA viral load (VL). We classified ART regimens according to the main antiretroviral class that composed the 3-drug regimen^29^: non-nucleoside reverse transcriptase inhibitors (NNRTIs), protease inhibitors (PIs), and integrase strand transfer inhibitors (INSTIs). We considered only ART used during TB treatment and our focus was on NNRTI (efavirenz) and INSTI (dolutegravir, raltegravir), as these regimens were recommended ART in Brazil for TB/HIV coinfection during the study period^29^. CD4 count was categorized as: <50 cells/µL, 50–200 cells/ µL, and ≥ 200 cells/µL. We considered VL as a categorical variable and, for the primary analysis, we defined VL as suppressed (< 1000 HIV-1 RNA viral copies/µL) and as non-suppressed (≥ 1000 HIV-1 RNA viral copies/µL). For the secondary analysis, we considered HIV virologic suppression as < 50 HIV-1 RNA viral copies/µL.

RePORT-Brazil genotyped 60 selected polymorphisms in 29 genes relevant to TB or HIV drug metabolism, and for this study, we selected *CYP2B6* and *UGT1A1*, which are associated with the metabolism of efavirenz and dolutegravir/raltegravir, respectively. Genotyping was done using MassARRAY® iPLEX Gold (Agena Bioscience™, California, USA) and Taqman (ThermoFisher Scientific, Massachusetts, USA).

### Statistical Analysis

Participant characteristics were described in the full study population and according to HIV-1 virologic suppression, summarizing continuous variables with median and interquartile range (IQR) and categorical variables with frequency and percentages.

Composite *CYP2B6* metabolizer genotype was defined based on combinations of three polymorphisms as follows: normal (1: 15582CC-516GG-983TT or 2: 15582CT-516GG-983TT); intermediate (3: 15582TT-516GG-983TT; 4: 15582CC-516GT-983TT; 5: 15582CC-516GG-983CT; 6: 15582CT-516GT-983TT; or 7: 15582CT-516GG-983CT); and slow (8: 15582CC-516TT-983TT; 9: 15582CC-516GT-983CT; 10: 15582CC-516GG-983CC) ^30^. And the *UGTA1A* metabolizer genotype was defined normal (887829CC), intermediate (887829CT), and slow (887829TT) ^31^. The normal genotypes for *CYP2B6* and *UGTA1A* are the inducers of the enzymatic system, and thus, metabolize ARV faster. ^14,25^ The outcome of interest was HIV-1 virologic suppression after at least two weeks of ART exposure.

We used Fisher’s exact test, and Z-score test to compare the outcomes proportions, grouping by ART class and SNP categories. Additionally, we performed a survival analysis to compare time-to-virologic suppression (with two different VL cutpoints), by ART groups and by SNP categories using the Wilcoxon-Gehan statistics. For this analysis, we considered the time-to-suppression variable in days since TB treatment start for ART-experienced participants and time since ART start for ART-naïve participants until up to six months after TB treatment ended. All analyses were performed using SPSS version 25.0, with a significance level of 0.05.

## Results

**Figure 1:**
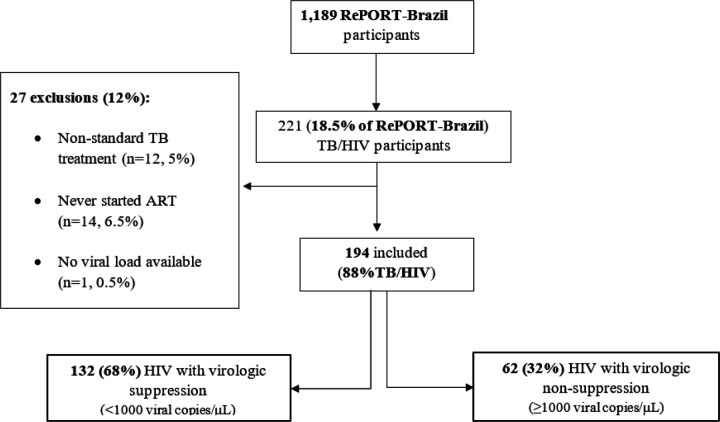
Study diagram

Among 1,189 participants with TB in RePORT-Brazil, 221 (18.5%) had HIV, and 194 (88%) were included in the analysis. Twelve participants (5%) used non-standard TB treatment, and 14 (6.5%) never started ART (Figure 1). In the primary analysis, virologic suppression was achieved in 68% of the participants (n=132). Overall, most of the participants were male (n=150, 77%), median age was 35 years (IQR 28–42), and 57% (n=111) were ART-naïve. The ART class most used was INSTI (51%), followed by NNRTI (40%) and PI (8%). Raltegravir was the most frequent INSTI (88%), while efavirenz, the most frequent NNRTI (99%). There were 46 (27%) and 82 (49%) participants with *CYP2B6* and *UGT1A1* normal (i.e., fastest) metabolizer genotypes, respectively (Table 1).

Irrespectively of the ART regimen, the overall virologic suppression was suboptimal (68%). Among participants with efavirenz-based ART, 36% (n=28) did not achieved virologic suppression, while among INSTI-based ART, 30% (n=30) did not achieved virologic suppression, with no significant difference in these proportions (p=0.34). Among them, the ones that received efavirenz-based ART were more likely to be *CYP2B6* normal metabolizers (n=8, 44%); and among persons treated with INSTI-based ART, the *UGT1A1* normal was also the most common (n=13, 50%) (Table 2).

**Figure 2:**
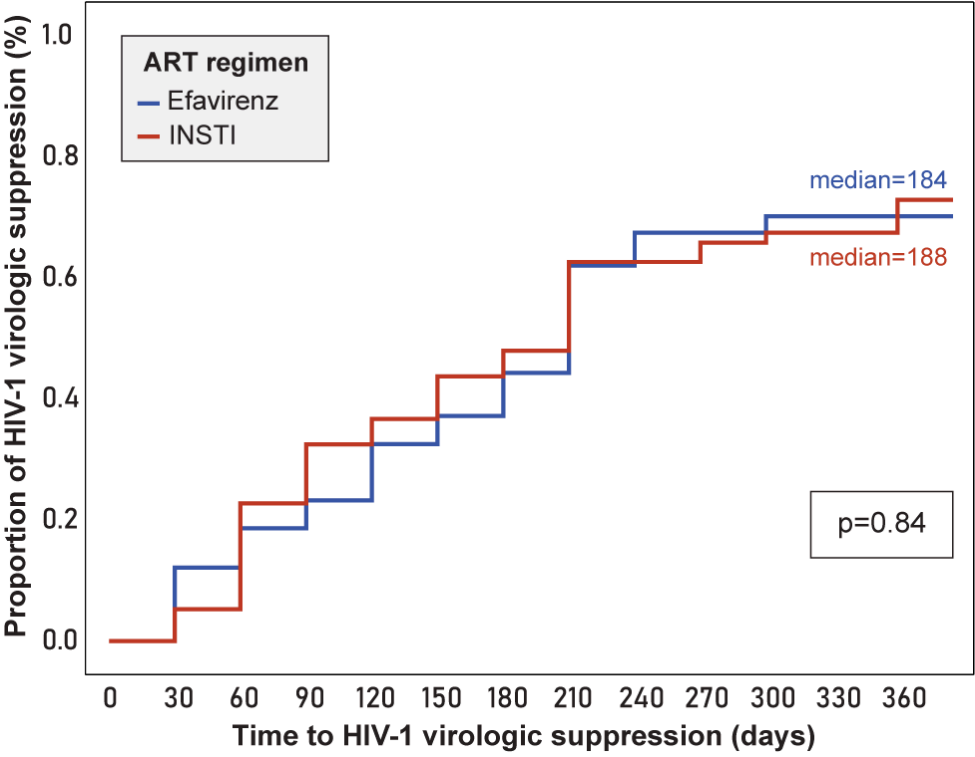
Time-to-HIV-1 virologic suppression (<1,000 viral copies/mL) in days, according to ART regimen (Efavirenz vs INSTI) Footnote: ART: antiretroviral therapy; INSTI: integrase strand transfer inhibitors: raltegravir or dolutegravir. Statistical test: Wilcoxon-Gehan test (survival analysis), significance level .05.

**Figure 3:**
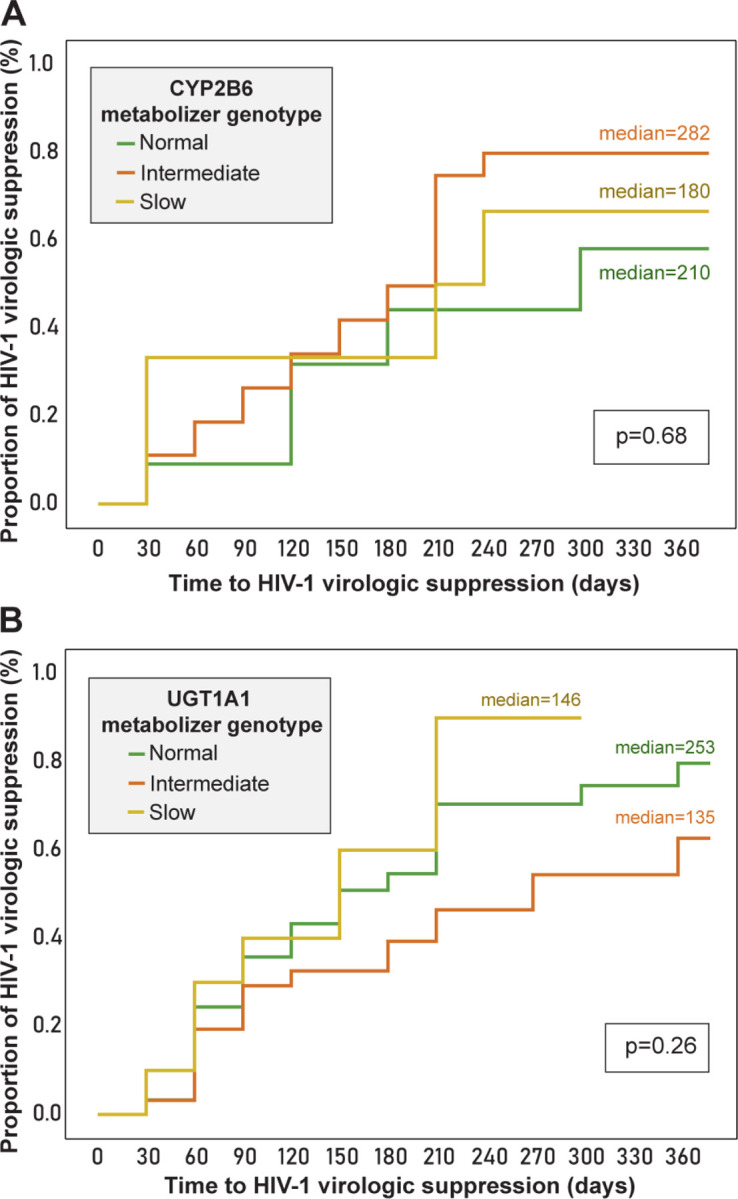
Time-to-HIV-1 virologic suppression (<1,000 viral copies/mL), according to (**A**) *CYP2B6* (Efavirenz) genotypes and (**B**) *UGT1A1* (INSTI) Footnote: ART: antiretroviral therapy; EFZ: efavirenz (a non-nucleoside reverse transcriptase inhibitor [NNRTI]); INSTI: integrase strand transfer inhibitors: raltegravir or dolutegravir. Statistical test: Wilcoxon-Gehan test (survival analysis), significance level .05.

The overall median time-to-virologic suppression, irrespective of efavirenz- or INSTI based ART, was 188 days (95% CI 172–203). For efavirenz-based ART, the median time was 184 days (95% CI 160–207), and for INSTI-based ART, 188 days (95% CI 144–231) (p=0.84) (Figure 2). Among efavirenz-based ART, considering the *CYP2B6* genotypes,the median time-to-suppression for normal, intermediate, and slow metabolizer profiles was 282 days, 180 days, and 210 days respectively (p=0.68). Among the INSTI-based ART, the *UGT1A1* normal metabolizer genotype had a median time-to-virologic suppression of 146 days, while intermediate of 253 days, and slow of 135 days (p=0.26) (Figure 3A and 3B respectively).

In the secondary analysis, lowering the VL cutpoint to <50 HIV-1 RNA viral copies/mL to define virologic suppression, more participants did not achieve the desire outcome. And among them, the proportion of normal metabolizers for *CYP2B6* and *UGT1A1* were again higher, although with no significance difference. The time-to-virologic suppression did not differ statistically among efavirenz-based and INSTI-based ART regimens and among *CYP2B6* and *UGT1A1* metabolizers genotypes (Supplemental Table 1, Supplemental Figure 1, and Supplemental Figure 2A and 2B).

## Discussion

We evaluated HIV-1 virologic suppression according to the genetic variants that affect NNRTI and INSTI metabolism among TB/HIV participants in Brazil. The virologic suppression was suboptimal, irrespective of efavirenz- vs INSTI-based ART regimens. Different from the literature,^32^ the time-to-virologic suppression between efavirenz- and INSTI-based regimens was similar, as well as between their metabolizer genotypes. However, among the ones not achieving virologic suppression, there was a higher proportion of *CYP2B6* and *UGT1A1* normal metabolizer genotypes, which is an expected finding.^30,33,34^ Moreover, we were expecting to find faster time-to-virologic suppression and higher rates of virologic suppression among INSTI-based regimens, compared to efavirenz-based regimens^32,35,36^, but no significant differences were found.

INSTI-based regimens were more commonly used than efavirenz-based regimens. This may be explained by a trend in recommendation for primary ART regimen for treatment-naïve TB/HIV patients in Brazil in 2017. Moreover, rates of resistance to efavirenz in the world and in Brazil is high – ranging from 3.4–5.5% and could exceed 10%^37^. This precludes using efavirenz-based regimens as a first line ART without baseline HIV-1 genotyping. ^38,39^ For that reason, since 2017, for TB/HIV patients meeting the criteria of severe disease – i.e., CD4 cell count < 100 cells/µL, disseminated TB, other concomitant opportunistic infection, and hospitalized patients –, the recommended ART regimen was to include raltegravir; and efavirenz-based treatment was recommended for TB/HIV without severe disease. However, since 2019, the first-line ART regimen recommended for TB/HIV participants has been a double dose dolutegravir-based ART regimen, expecting to increase effectiveness as dolutegravir is a safe and well tolerated drug, with a higher genetic barrier to resistance.^40^ Raltegravir is no longer recommended as an option for TB/HIV patients.^9,41^ Efavirenz-based regimens are also well-tolerated, but neuropsychiatric adverse reactions, and primary and acquired resistance, can limit its use.^42,43^

Regarding the genetic variants, we found higher proportions of *UGT1A1* and *CYP2B6* normal metabolizer genotypes among participants not achieving virologic suppression. And this is expected as the faster the ART is metabolized, the lower will be its bioavailability, leading to lower drug exposure, and ultimately not suppressing the HIV-1 VL. This finding suggests an association, that could be stronger if there were a larger sample size.

Our study had limitations. We were unable to handle the VL as a continuous variable because not all participants had VL measured at the same timepoint during the follow up. Not all participants had SNPs available, and we did not evaluate HIV-1 treatment toxicity. Conversely, we highlight that we had a prospective cohort study that is representative of the Brazilian population^26^, with important data on human genetic variants and ART, as well as treatment outcomes, and *UGT1A1* and *CYP2B6* metabolizer profiles.

In this observational cohort of patients treated for TB/HIV, the proportion of participants achieving virologic suppression was low, and genetic variants affecting ART metabolism were not significantly associated with the likelihood of virologic suppression.

## Supplementary Material

Supplement 1 Tables 1 to 2 are available in the Supplementary Files section

## Data Availability

The datasets used and analyzed during the current study are available from the corresponding author on reasonable request.
