# Supplementary material for: Pharmacogenomic associations with HIV-1 virologic suppression in TB/HIV patients
Source: Res Sq. 2024 Dec 16:rs.3.rs-5418156. Preprint. [Version 1] doi: 10.21203/rs.3.rs-5418156/v1 (PMC11702782; doi:10.21203/rs.3.rs-5418156/v1)
Supplement: Supplement 1 — Tables 1 to 2 are available in the Supplementary Files section [file NIHPPRS5418156v1-supplement-1.pdf]

Supplementary Files

This is a list of supplementary files associated with this preprint. Click to download.

- BMCIDSupplementaryMaterial.docx
- Tables.docx
